# Editorial: AI and big data integration in orthopedic regenerative medicine

**DOI:** 10.3389/fcell.2026.1892774

**Published:** 2026-06-10

**Authors:** Zhen Yang, Zhiguo Yuan, Xiaohao Wu, Jianjing Lin

**Affiliations:** 1 Arthritis Clinical and Research Center, Peking University People’s Hospital, Beijing, China; 2 Arthritis Institute, Peking University, Beijing, China; 3 Department of Bone and Joint Surgery, Renji Hospital, School of Medicine, Shanghai Jiao Tong University, Shanghai, China; 4 Immunology and Rheumatology, School of Medicine, Stanford University, Palo Alto, CA, United States; 5 Department of Sports Medicine and Rehabilitation, Peking University Shenzhen Hospital, Shenzhen, China

**Keywords:** artificial intelligence, big data, biomaterials, clinical translation, multimodal data integration, orthopedics, regenerative medicine, tissue engineering

Orthopedic disorders such as osteoarthritis, intervertebral disc degeneration, osteoporosis, and large bone or cartilage defects represent a rising global burden in an aging society (GBD, 2021 Osteoarthritis Collaborators, 2023; Hunter and Bierma-Zeinstra, 2019). Regenerative medicine and tissue engineering offer transformative strategies, yet clinical translation remains constrained by biological heterogeneity, fragmented evidence, and the inherent complexity of biomaterial development (Langer and Vacanti, 1993; Murphy et al., 2020). Artificial intelligence (AI) and big data are now reshaping this landscape, evolving from auxiliary analytic tools into a connective infrastructure that integrates mechanism, material, and medicine (Topol, 2019; Acosta et al., 2022). This Research Topic brings together eight contributions that collectively envision how AI may accelerate the next phase of orthopedic regeneration.

A first conceptual axis concerns AI-enabled design and translation. Xu et al. delineate how AI supports biomaterial screening, tissue construct design, surgical assistance, and post-operative assessment across the translational chain. Building on this view, Liu M. et al. describe AI-driven biomaterial design as an intelligent closed loop linking reverse design, performance prediction, and biological response. Such paradigms mirror broader advances in materials informatics and autonomous experimentation, where computational prediction and high-throughput validation are progressively unified (Butler et al., 2018; Stach et al., 2021). The implication for orthopedics is significant: future scaffolds, hydrogels, and combination products may be designed within iterative AI–experiment loops rather than through empirical optimization alone.

A second axis addresses intervertebral disc repair, a mechanically and biochemically demanding regenerative target (Sakai and Andersson, 2015). Wang et al. reconstruct the evidence architecture of hydrogel-based disc repair through AI-assisted mapping, clarifying knowledge clusters and unresolved gaps. Shi and Wang complement this with an opinion proposing an AI-driven blueprint to decode disc repair mechanisms and guide intelligent biomaterial design. Together, they suggest that future disc regeneration strategies will rely on the convergence of literature-scale evidence synthesis, mechanistic modeling, and computationally informed material engineering.

A third axis concerns multimodal data and knowledge integration. Ding et al. review how AI bridges imaging, omics, and clinical data to enable precise stratification and individualized regenerative strategies—a trajectory consistent with the broader rise of multimodal biomedical AI (Acosta et al., 2022). Li et al. apply AI- and big data-driven knowledge mapping to exosome–hydrogel research, revealing emerging hotspots at the interface of cell-free therapy and biomaterials (Riau et al., 2019). These contributions highlight that future orthopedic regeneration will increasingly depend on the orchestration of heterogeneous data—from molecules to patient outcomes.

A fourth axis explores AI for clinical reasoning and mechanism discovery. Xiao et al. benchmark conversational AI systems for evidence-based decision-making in cartilage repair, reflecting both the promise and the risks of large language models in medicine (Singhal et al., 2023). At the molecular level, Liu D. et al. identify shared lactylation-related gene signatures between osteoporosis and chronic kidney disease, extending orthopedic regeneration into systemic metabolic and inflammatory contexts (Zhang et al., 2019). Together, these works signal a future in which AI not only informs material design but also reshapes clinical decisions and reveals previously hidden disease axes.

Looking forward, several priorities emerge. First, the field requires standardized, multi-center, and longitudinal datasets that can support robust model training and external validation. Second, interpretable and mechanistically grounded AI will be essential, particularly when models guide biomaterial selection or clinical interventions. Third, computational predictions must be coupled with prospective experimental and clinical validation to ensure translational reliability. Fourth, ethical, regulatory, and human-factor considerations must evolve in parallel with technical capability, especially as generative and agentic AI systems enter clinical workflows. Finally, the most transformative advances will likely emerge from genuinely interdisciplinary teams uniting orthopedic surgeons, stem cell biologists, materials scientists, bioinformaticians, engineers, and data scientists.

AI should not be regarded as a replacement for biological insight or clinical judgment, but as a connective infrastructure that converts complex data into testable hypotheses, optimized biomaterials, personalized interventions, and measurable patient outcomes. The contributions assembled here outline an emerging blueprint for this convergence and point toward a future in which orthopedic regenerative medicine is increasingly data-informed, mechanism-aware, and translationally accelerated. We hope this Research Topic catalyzes further interdisciplinary innovation and supports the responsible integration of AI and big data technologies into the next-generation of orthopedic care.
